# Insulin signaling mediates previtellogenic development and enhances juvenile hormone-mediated vitellogenesis in a lepidopteran insect, *Maruca vitrata*

**DOI:** 10.1186/s12861-019-0194-8

**Published:** 2019-07-05

**Authors:** Md. Abdullah Al Baki, Dae-Weon Lee, Jin Kyo Jung, Yonggyun Kim

**Affiliations:** 1 Department of Plant Medicals, Andong National University, Andong, 36729 Korea; 2 School of Chemistry and Life Sciences, Kyungsung University, Busan, 48434 Korea; 3 Division of Crop Cultivation and Environment Research, Department of Central Area Crop Science, National Institute of Crop Science, Rural Development Administration, Suwon, 16429 Korea

**Keywords:** Insulin-like peptide, Reproduction, Oocyte, Vitellogenesis, *Maruca vitrata*

## Abstract

**Background:**

Insulin/insulin-like growth peptide signaling (IIS) down-regulates hemolymph sugar level and facilitates larval growth in the soybean pod borer, *Maruca vitrata*. The objective of this study is to determine whether IIS of *M. vitrata* can mediate ovarian development of adult females.

**Results:**

A pair of ovaries consists of 8 ovarioles, each of which is separated into distal germarium and proximal vitellarium in *M. vitrata*. In the germarium, oocyte development occurred with active mitotic activity which was visible by incorporating bromodeoxyribose uridine. Previtellogenic development and subsequent vitellogenesis began soon after adult emergence. They continued with increase of female age. Oocyte development was facilitated by up-regulation of vitellogenin (Vg) and Vg receptor (VgR) gene expression. Larval diets significantly influenced on ovarian development of *M. vitrata* because oocyte development varied with pupal size derived from larvae treated with different nutritional diets. Its ovarian development was dependent on endocrine signal(s) from the head because decapitation soon after adult emergence prevented oogenesis and subsequent vitellogenesis along with marked reduction of *Vg* and *VgR* expression. Topical application of juvenile hormone (JH) significantly recovered its ovarian development whereas farnesoic acid (a precursor of JH biosynthesis) or 20-hydroxyecdysone treatment did not. JH stimulated vitellogenesis and choriogenesis, but not previtellogenic development. In contrast, insulin injection to decapitated females stimulated oocyte differentiation and vitellogenesis along with increase of *Vg* and *VgR* expression. To further analyze the effect of insulin on ovarian development, expression of four IIS components (InR, FOXO, Akt, and TOR) genes was manipulated by RNA interference. Hemocoelic injection of gene-specific double stranded RNAs significantly reduced their target gene mRNA levels and interfered with ovarian development. An addition of insulin to JH treatment against decapitated females enhanced the gonadotropic effect of JH by stimulating oogenesis.

**Conclusions:**

IIS plays crucial role in mediating previtellogenic development of *M. vitrata* in response to nutrient signal. It also enhances the gonadotropic effect of JH II on vitellogenesis.

**Electronic supplementary material:**

The online version of this article (10.1186/s12861-019-0194-8) contains supplementary material, which is available to authorized users.

## Background

High reproductive potential is a biological character of insects [1]. Social insects such as honey bee and termite queens are well known to have a huge number of egg production and subsequent oviposition [2]. Egg production of female insects is a sequential process consisting of previtellogenic development, vitellogenesis, and choriogenesis [3, 4]. Previtellogenic development represents the formation of oocytes from oogonial stem cells by mitosis and meiosis. It occurs in the distal part of each ovariole [5]. Vitellogenesis is the process of accumulating vitellogenin (Vg) and other biomaterials into growing oocytes [6, 7]. After oocytes are fully grown, they are coated with chorion by follicular epithelium to become “eggs” in the proximal part of ovarioles [8]. These eggs are then ovulated to oviducts and fertilized just before oviposition.

Different endocrine signals are associated with ovarian development in insects [9]. Juvenile hormone (JH) is a sesquiterpenoid that mediates a status quo effect during immature stage to prevent precocious metamorphosis [10, 11]. However, in adults, it stimulates ovarian development as gonadotropin in various insects [12–14]. JH directly stimulates Vg biosynthesis in *Manduca sexta* and *Locusta migratoria* [15, 16]. In mosquito females, it has endocrine action of 20-hydroxyecdysone (20E) [17, 18]. JH usually facilitates Vg uptake of growing oocytes by inducing follicular patency [19–21]. Thus, any inhibition of JH action can lead to severe impairment of ovarian development.

Insulin-like peptides (ILPs) are known to mediate ovarian development in some insects [22]. In *Drosophila*, ILPs can stimulate oogonial proliferation to produce oocytes in the stem cell niche located in the germarium of the distal ovariole [23]. Nutrient signal derived from reserves accumulated during larval period stimulates the brain to produce specific ILP(s) [24, 25]. Like vertebrate relaxin, the produced ILP stimulates ovarian development through a common insulin receptor (InR) and initiates insulin/insulin-like growth factor signal (IIS) which is highly conserved among animals [26]. Especially, four IIS components (InR, serine-threonine protein kinase (Akt), Forkhead Box O (FOXO), and target of rapamycin (TOR)) have been assessed in physiological functions in controlling hemolymph sugar level and larval development [27, 28].

The legume pod borer, *Maruca vitrata* (Lepidoptera: Crambidae), is distributed in subtropical and tropical regions. It damages several leguminous crops with losses in the range of 20–80% [29]. Economic damage caused by *M. vitrata* may be explained by its high fecundity. *M. vitrata* is known to lay a lot of eggs (about 500 eggs per female), causing outbreaks under favorable conditions. *M. vitrata* females contain matured eggs before mating and oviposit soon after mating in the presence of stimulant from host floral volatiles [30]. Fecundity is one of the characters used by biologists to investigate individual fitness. It may greatly vary depending on the species and its life cycle [31]. It is also affected by a series of abiotic (e.g., temperature) and biotic (e.g., nutritional status, mating status, and age) parameters. It has been shown that fecundity is positively correlated with the number of ovarioles containing oocytes [32]. Thus, the high reproductive potential of *M. vitrata* can be understood through physiological analysis of ovarian development.

This study analyzed ovarian development of *M. vitrata* with respect to endocrine signals. Its ovarian development is known to be correlated with nutrients reserved during larval stage [33]. Thus, physiological role of IIS in its adult reproduction was investigated. This study also tested a functional synergism of IIS with JH signal in ovarian development of *M. vitrata*.

## Results

### Ovarian development of *M. vitrata* females

**Fig. 1:**
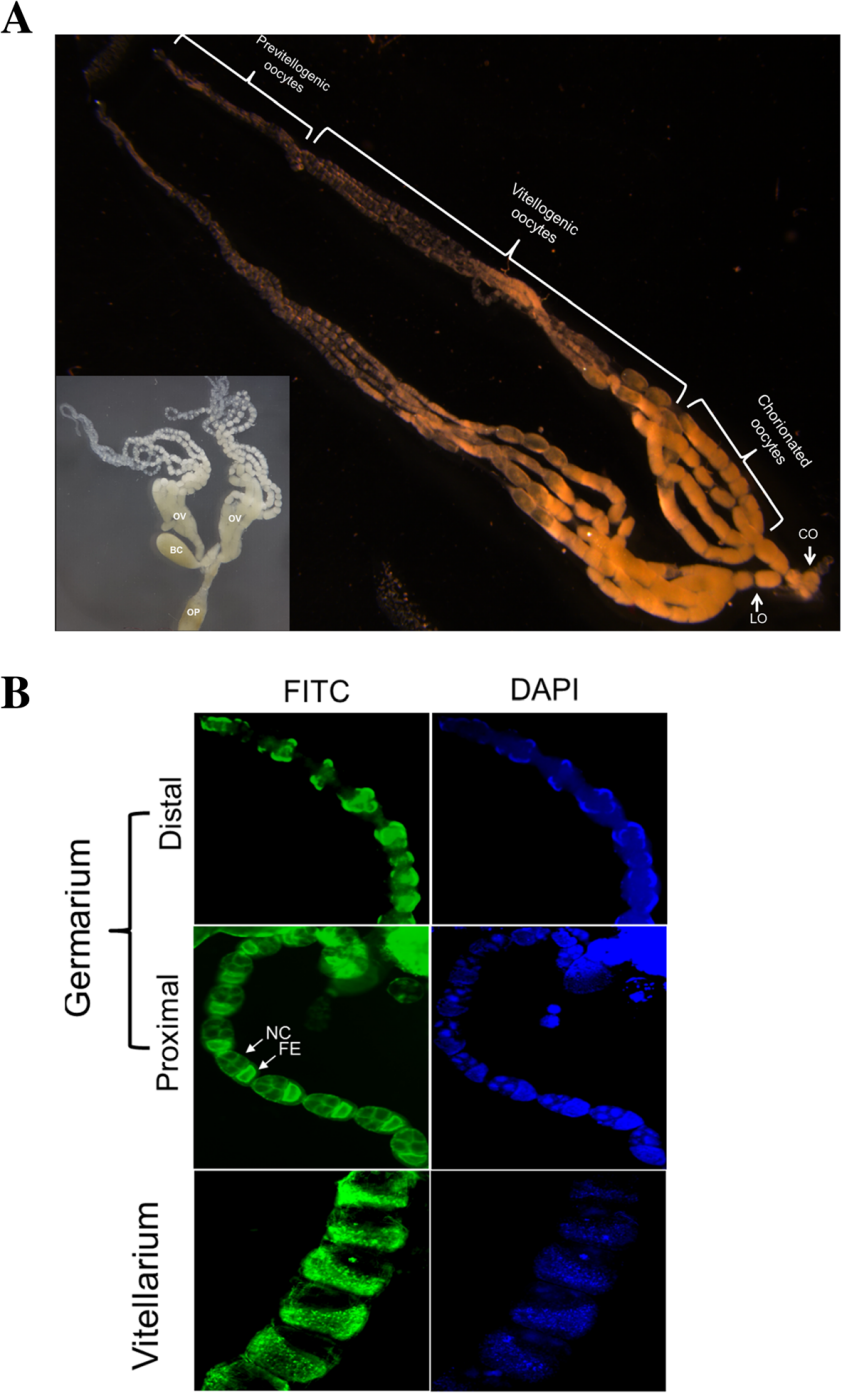
Ovary structure of *M. vitrata* females. **a** Total ovary (OV) structure of 5 days old female. A pair of ovaries is extended from common oviduct (CO) via lateral oviduct (LO). In each of ovariole, oocyte development is subdivided into previtellogenic oocyte, vitellogenic oocyte, and chorionated oocyte under a stereomicroscope. Inset figure shows bursa copulatrix (BC) and ovipositor (OP) near ovaries. **b** Comparison of oocyte development between germarium and vitellarium. In germarium, oocytes are invisible in distal region. However, they are distinct in the proximal region along development of nurse cell (NC) and follicular epithelium (FE). In vitellarium, oocyte increases with increase of FE area. F-actin filament is specifically recognized by FITC-tagged phalloidin (green) while nucleus is stained with DAPI (blue). Cells are observed under a fluorescence microscope at 200 × magnification

At 3 days of emergence, both ovaries of virgin females were well developed in size (Inset figure of Fig. 1a). Each ovary contained four ovarioles. Each ovariole was divided into three parts: previtellogenic (PV), vitellogenic (VT), and chorionated (CH) eggs (Fig. 1a). PV contained oocytes before vitellogenesis, in which oocytes were uniform in size. VT contained oocytes under vitellogenesis, in which oocytes increased in size along with ovariole to the proximal region. CH contained oocytes covered with chorion.

Distal region of the ovariole contained cells before oocyte differentiation. Oocytes were visible in previtellogenic region and surrounded by follicular epithelium (Fig. 1b). Nurse cells were neighboring to oocytes, indicating polytrophic ovarioles of *M. vitrata*. In vitellogenic region, oocytes grew in size along with increase of follicular epithelium area.

At the most distal region of each ovariole, undifferentiated cells were highly detected by BrdU staining, indicating active cell division (Additional file 1: Figure S1A). Subsequently, a series of cell division with increasing number of nuclei (see DAPI staining) was detected (Additional file 1: Figure S1B). At the end of this cell division, a follicle containing nurse cells and an oocyte surrounded by follicular epithelium were observed (Additional file 1: Figure S1C).

### Expression profiles of vg and VgR in *M. vitrata* females

**Fig. 2:**
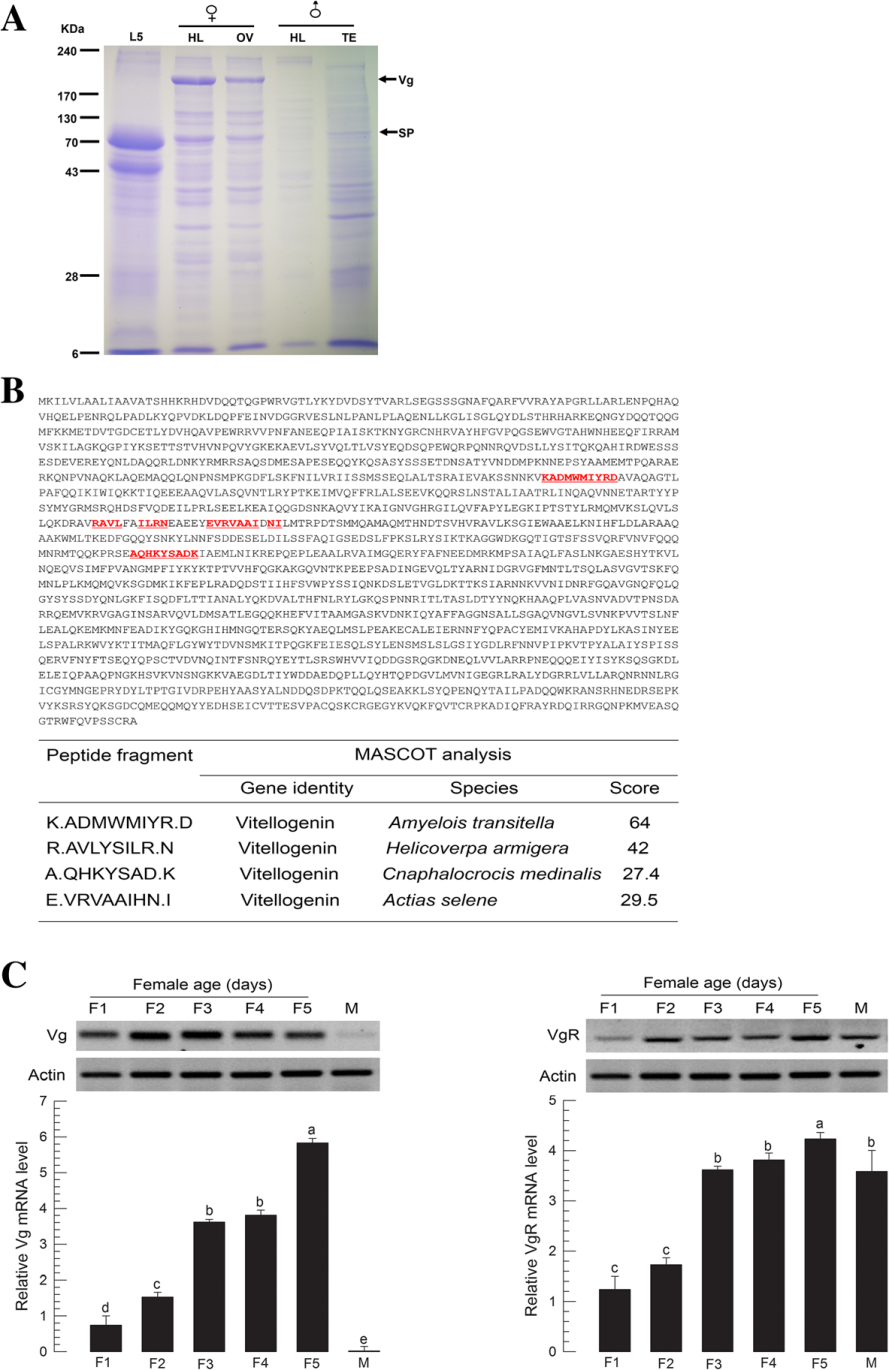
Identification and expression profile of vitellogenin (Vg) and vitellogenin receptor (VgR) of *M. vitrata*. **a** Vg on 10% SDS-PAGE. Vg protein was identified in 5 days old adult female hemolymph (HL) and ovary (OV), but not in male HL, testis (TE), or L5 larvae. L5 larval HL contained a large amount of storage protein (SP). **b** LC-MS/MS analysis of Vg protein band and its MASCOT prediction. The sequence of *M. vitrata* Vg (GenBank accession number: MG799570) contains fragments identified by LC-MS/MS. **c** Expression analyses of *Vg* and *VgR* in females at different ages (1–5 days after emergence) and males (5 days old after emergence) using RT-PCR (gel picture) and RT-qPCR (graph). All treatments in RT-qPCR were independently replicated three times. *β-Actin* expression was used as reference in RT-qPCR to normalize target gene expression level. Different letters above standard deviation bars indicate significant difference among means at Type I error = 0.05 (LSD test)

To analyze vitellogenesis of *M. vitrata,* vitellogenin (Vg) and Vg receptor (VgR) genes were identified and their expression levels were monitored along with female development (Fig. 2). Vg protein was detected in female adults, but not detected in larval hemolymph or male adult in SDS-PAGE (Fig. 2a). Its apparent size on the protein gel was approximately 200 kDa. LC-tandem MS analysis revealed that the Vg band was highly matched to other lepidopteran Vg proteins (Fig. 2b). Vg and VgR genes were predicted (Additional file 2: Figure S2, Additional file 3: Figure S3) from a transcriptome of *M. vitrata* (GenBank accession numbers: MG799570 for Vg and MG799569 for VgR). Open reading frame (ORF) of Vg encoded 1777 amino acids with molecular weight of 202.06 kDa and pI at 8.27. VgR ORF encoded 1798 amino acids with molecular weight of 198.28 kDa and pI at 4.98. RT-PCR analysis indicated that *Vg* and *VgR* were expressed in females. Their expression levels increased with age (Fig. 2c). *Vg* expression was female-specific while *VgR* was expressed in both sexes.

### Influence of larval diet on adult ovarian development

**Fig. 3:**
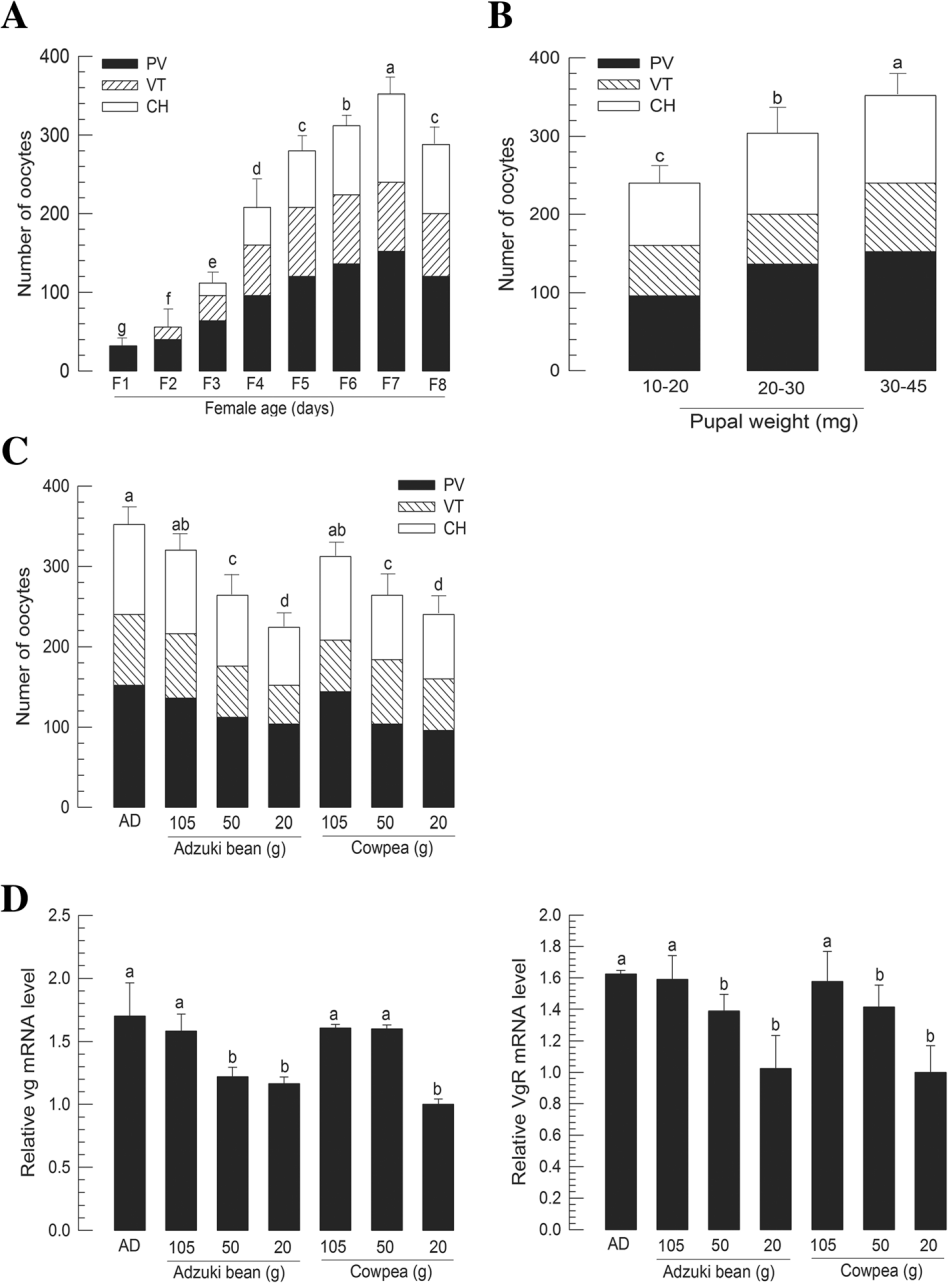
Effect of larval nutrients on adult ovarian development of *M. vitrata*. **a** Oocyte development according to female age. Ovarioles were separated from different aged females (1–8 days after emergence) to count the number of previtellogenic oocytes (PV), vitellogenic oocytes (VT), and chorionated (CH) oocytes. For each age treatment, 10 females were analyzed. **b** Influence of pupal weight on ovarian development. Ten females (5 days old after emergence) were randomly collected from three body weight pupal groups and assessed in oocyte development. **c** Effect of larval diets on oocyte development. A standard artificial diet (‘AD’), three adzuki bean diets in different nutritional amounts, and three cowpea diets in different nutritional amounts were assessed for this analysis. For each diet treatment, 10 females (5 days old after emergence) were randomly selected and their oocyte development was assessed. **d** Expression levels of *Vg* and *VgR* in adults developed from larvae treated with different diets. Expression levels of these two gene were quantified by RT-qPCR in 5 days old females. All treatments were independently replicated three times. *β-Actin* expression was used as reference gene of RT-qPCR to normalize target gene expression level. Different letters above standard deviation bars indicate significant difference among means at Type I error = 0.05 (LSD test)

In virgin females, total number of oocytes increased with adult age until 7 days old (Fig. 3a). The increase in the number of oocytes was accompanied by an increase of the number of PV oocytes. To determine whether oocyte development of adult females was affected by larval diet, different nutritional diets were fed to larvae and oocyte development was analyzed for the resulting female adults (5 days old). Various diet treatments produced different body weights of pupae. Total numbers of oocytes in adult females were increased with increase of pupal weights (Fig. 3b). There was significant difference (*P* < 0.05) in the number of previtellogenic oocytes among larval diet treatments. To see more detailed correlation between larval diets and adult oocyte development, numbers of oocytes formed in adult females were compared among larval diets (Fig. 3c). The nutritional quality of larval diet was directly related to oocyte development of adult females. Again, larval diets influenced oogenesis because the number of previtellogenic oocytes was different according to diet quality. In addition, larval diet quality influenced Vg production. Moreover, expression levels of *Vg* and *VgR* in adult females were significantly (*P* < 0.05) different among different groups of larval diets (Fig. 3d).

### Effect of decapitation and JH on ovarian development

**Fig. 4:**
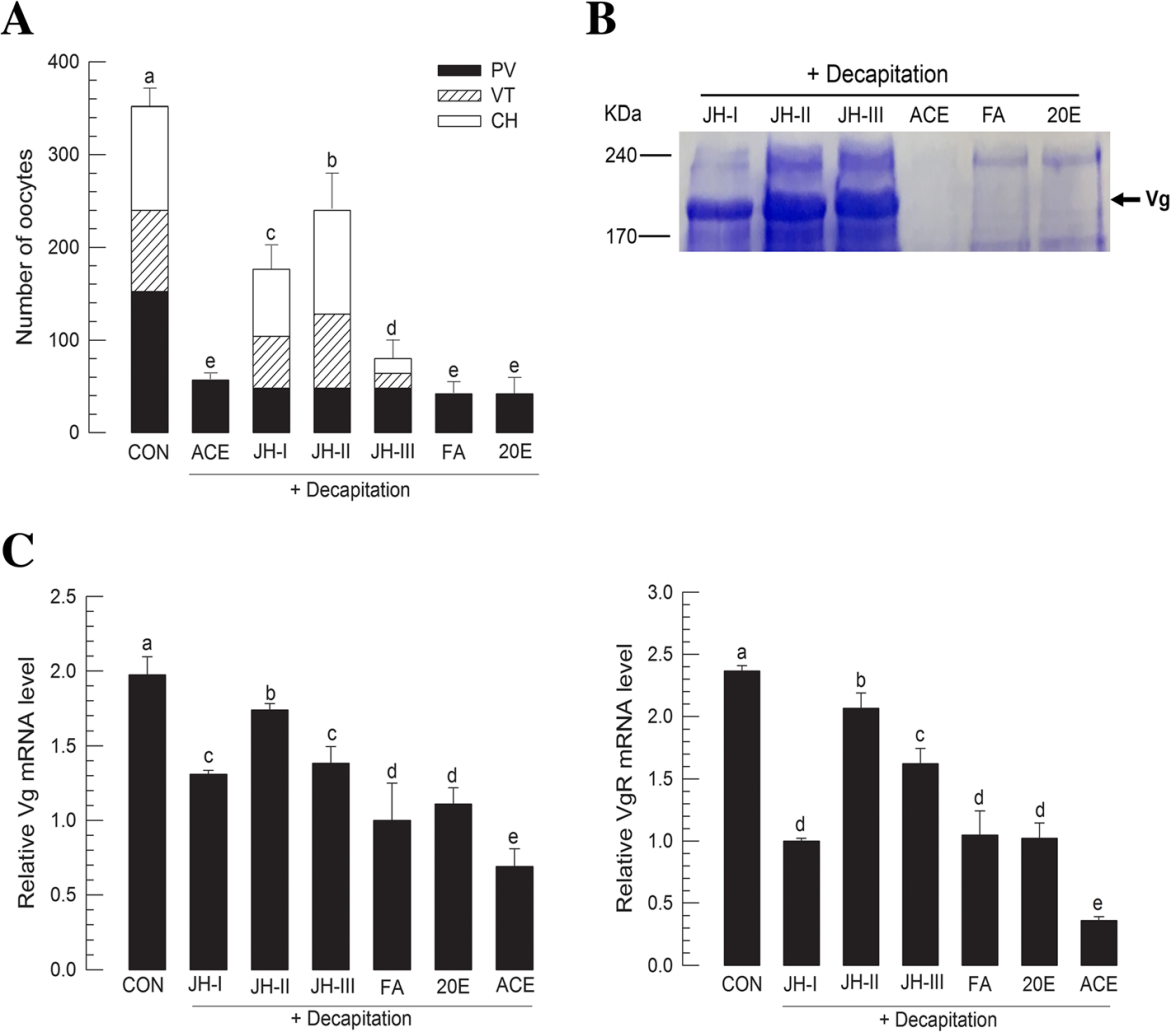
Effect of juvenile hormone (JH) on ovarian development of *M. vitrata*. **a** Effect of decapitation and subsequent hormonal treatment on oocyte development. Decapitation was performed in teneral female adults soon after emergence (< 12 h). Hormones were injected into decapitated females in a concentration of 1 μg per female using a microsyringe. Three JHs (JH I, JH II, and JH III), farnesoic acid (FA), and 20-hydroxyecdysone (20E) were assessed. Acetone (‘ACE’) was used as a control in decapitation treatment. ‘CON’ represents females without decapitation treatment. After 5 days at 25 °C, 10 females in each treatment were assessed for oocyte development. **b** Vitellogenin (Vg) production analysis using 10% SDS-PAGE. Hemolymph was collected from 5 days old females treated with different hormones. **c** Expression levels of *Vg* and *VgR* in adults treated with different hormones. Expression levels of these two genes were quantified by RT-qPCR in 5 days old females. All treatments were independently replicated three times. *β-Actin* expression was used as a reference in RT-qPCR to normalize target gene expression level. Different letters above standard deviation bars indicate significant difference among means at Type I error = 0.05 (LSD test)

To understand endocrine signal(s) from the brain to control ovarian development, decapitation was applied to teneral female adults (Fig. 4). Soon after adult emergence (< 4 h), females were decapitated and reared at 25 °C for 5 days. Decapitated females did not produce any vitellogenic oocytes. They had much lower number of previtellogenic oocytes compared to control ones. Different developmental hormones were then applied to these decapitated female adults to determine gonadotropin of *M. vitrata* (Fig. 4a). Application of JHs stimulated egg production. However, treatment with 20E or JH precursor (farnesoic acid: FA) did not rescue the decapitation effect on ovarian development. Among JHs, JH II was significantly (*P* < 0.05) superior to JH I and JH III in egg production. Although JH treatment increased the number of vitellogenic oocytes, it did not increase the number of previtellogenic oocytes.

The effect of JH on ovarian development was also confirmed by analyzing Vg protein level in female hemolymph (Fig. 4b). Vg protein was detected in females from three JH (JH I to III) treatment groups, but not in the control, FA, or 20E treatment group. To support this protein expression result, mRNA levels of *Vg* and *VgR* were analyzed by RT-qPCR (Fig. 4c). Expression levels of both genes were highly induced by JH treatments, but not by FA and 20E treatments, compared to the level of acetone-treated decapitated females.

### Influence of IIS on ovarian development

**Fig. 5:**
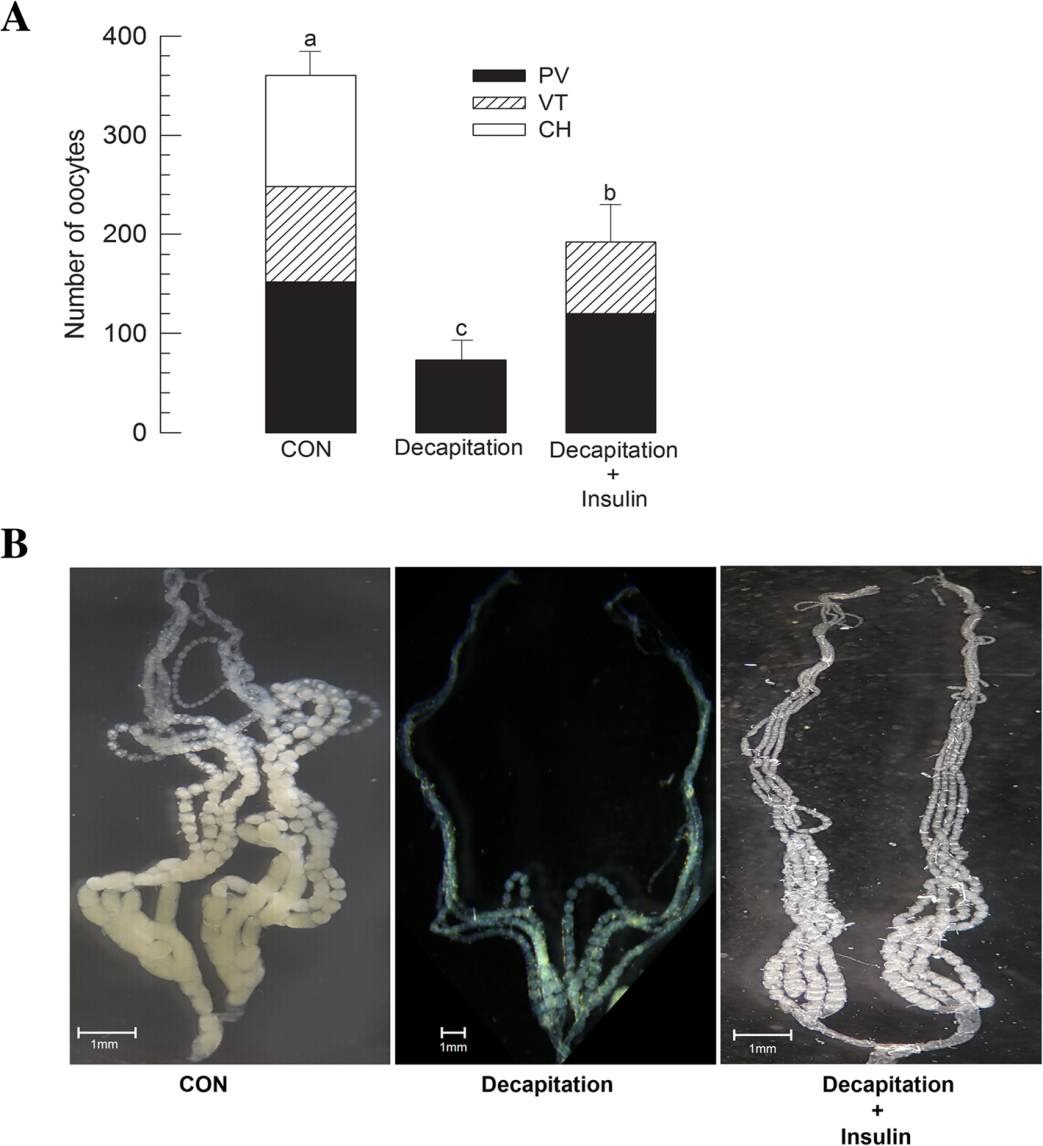
Effect of insulin on ovarian development of *M. vitrata*. **a** Effect of decapitation and subsequent insulin treatment on oocyte development. Decapitation was performed in teneral female adults soon after emergence (< 12 h). Porcine insulin was injected into decapitated females in a concentration of 1 μg per female using a microsyringe. ‘CON’ represents females without decapitation treatment. After 5 days at 25 °C, 10 females in each treatment were assessed for oocyte development. Different letters above standard deviation bars indicate significant difference among means at Type I error = 0.05 (LSD test). **b** Photos showing ovaries from females treated with decapitation or insulin addition compared to untreated (CON) female ovary. Scale bar represents 1 mm

To explore previtellogenic development stimulated by endocrine signal, insulin signaling was assessed by adding porcine insulin to decapitated females (Fig. 5). Porcine insulin is known to be effective in regulating larval growth and hemolymph sugar level of *M. vitrata* [27, 34]. In the present study, the addition of porcine insulin significantly (*P* < 0.05) rescued previtellogenic development (Fig. 5a). The addition of porcine insulin also stimulated vitellogenesis. However, it did not induce choriogenesis (Fig. 5b).

**Fig. 6:**
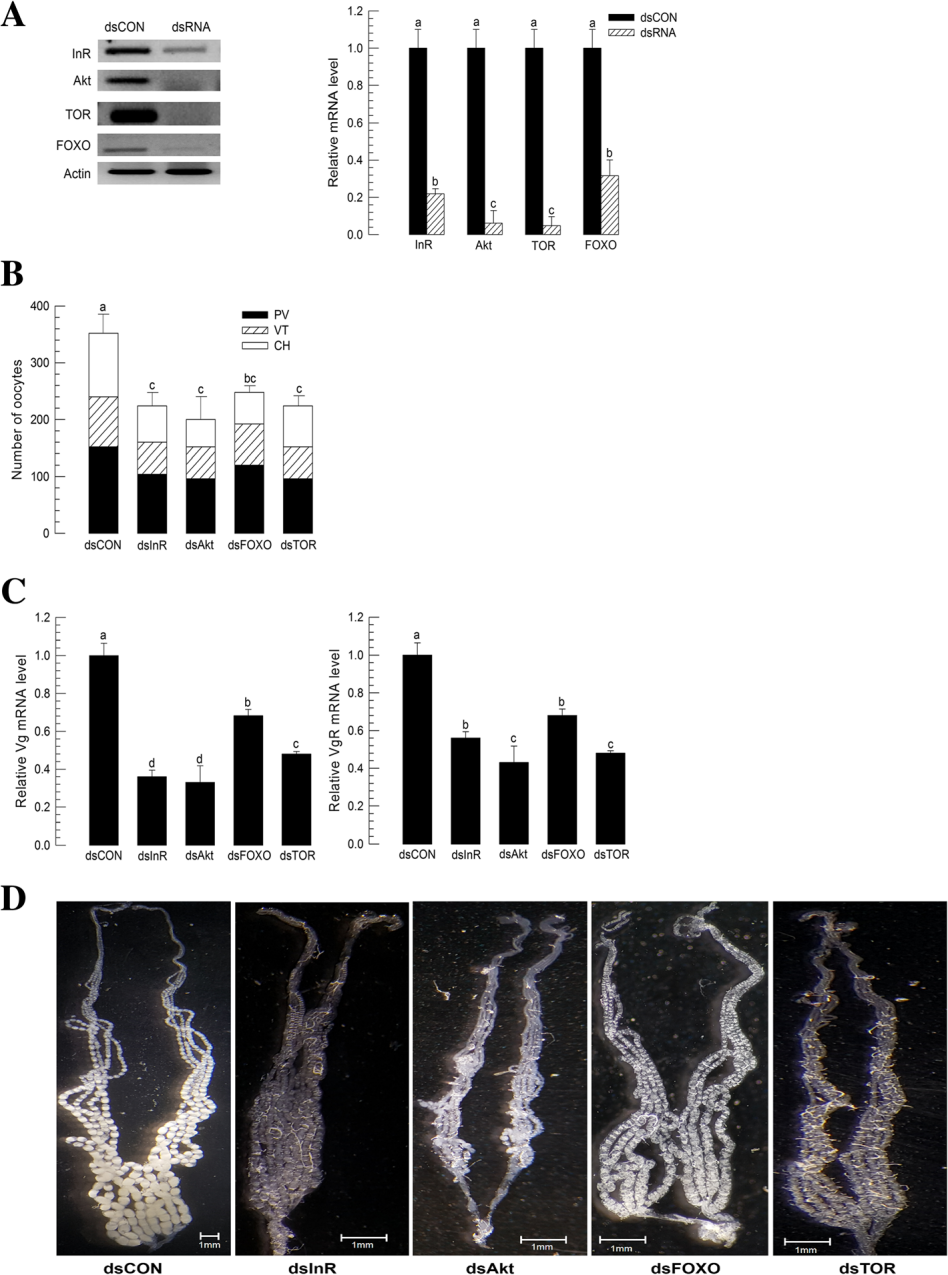
Influence of insulin-like peptide/IGF signaling (IIS) on ovarian development of *M. vitrata*. **a** RNA interference (RNAi) using dsRNAs specific to insulin receptor (InR), serine/threonine-protein kinase (Akt), target of rapamycin (TOR), and Forkhead box protein O (FOXO). dsRNA (1 μg) specific to each gene was injected to 5 days old pupae (pharate adult stage). Newly emerged adults were reared with 10% sugar until 5th day. Changes in mRNA levels were monitored by RT-qPCR using β-actin gene expression as reference to normalize target gene expression level. Control RNAi (dsCON) used a viral gene, CpBV302, by injecting its dsRNA at the same dose. All treatments were independently replicated three times. Different letters above standard deviation bars indicate significant difference among means at Type I error = 0.05 (LSD test). **b** RNAi effect of IIS components (InR, Akt, FOXO or TOR) on oocyte development. dsInR, dsAkt, dsFOXO, and dsTOR represent specific respective dsRNAs. Ovarioles were separated from 5 days old females to count the number of previtellogenic oocytes (PV), vitellogenic oocytes (VT), and chorionated (CH) oocytes. For each treatment group, 10 females were analyzed. **c** Expression levels of *Vg* and *VgR* in adults treated with different dsRNAs. Expression levels of these two genes were quantified by RT-qPCR in 5 days old females. All treatments were independently replicated three times. *β-Actin* expression was used as reference in RT-qPCR to normalize target gene expression level. Different letters above standard deviation bars indicate significant difference among means at Type I error = 0.05 (LSD test). **d** Photos showing ovaries from females treated with different dsRNAs. Scale bar represents 1 mm

To further investigate the effect of insulin on oocyte differentiation, expression levels of four IIS components (InR, FOXO, Akt, and TOR) were altered by RNAi treatment (Fig. 6). Injecting dsRNAs specific to IIS component genes significantly (*P <* 0.05) suppressed their gene expression levels (Fig. 6a). RNAi efficiencies for all four IIS components ranged from 68 to 90%. Under these RNAi conditions, ovarian development was significantly (*P* < 0.05) reduced (Fig. 6b). These RNAi treatments also suppressed gene expression levels of *Vg* and *VgR* (Fig. 6c), resulting in hypotrophied ovaries (Fig. 6d).

### Cooperative effect of insulin and JH on ovarian development

**Fig. 7:**
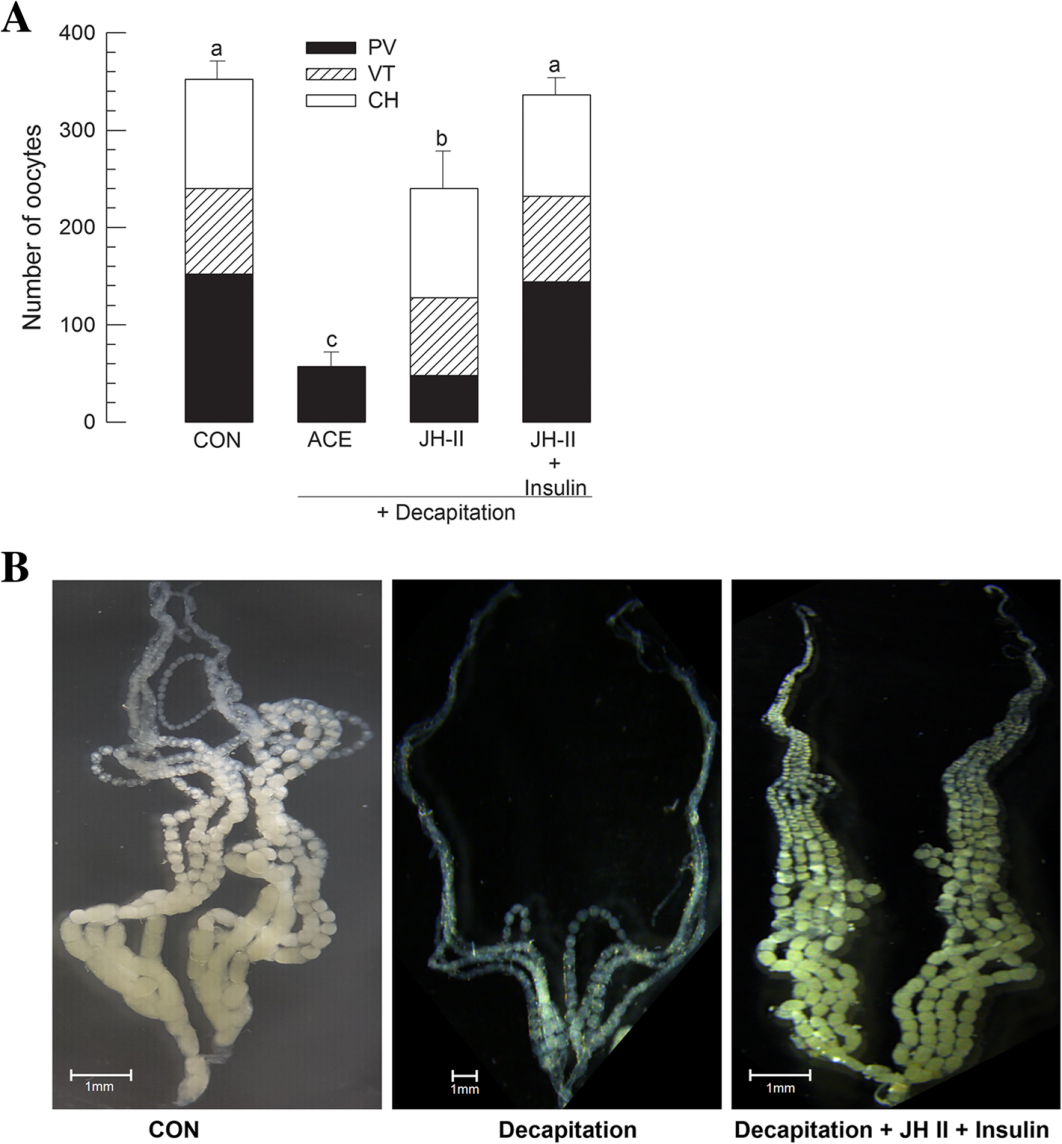
Synergistic effect of insulin and JH II on ovarian development of *M. vitrata*. **a** Effect of decapitation and subsequent hormone treatment on oocyte development. Decapitation was performed in teneral female adults soon after emergence (< 12 h). JH II or porcine insulin was injected into decapitated females at 1 μg per female using a microsyringe. ‘CON’ represents females without decapitation treatment. After 5 days at 25 °C, 10 females in each treatment were assessed for oocyte development. Different letters above standard deviation bars indicate significant difference among means at Type I error = 0.05 (LSD test). **b** Photos showing ovaries from females treated with decapitation or hormone addition compared to untreated (CON) female ovary. Scale bar represents 1 mm

JH stimulated vitellogenesis, but not oocyte differentiation while IIS stimulated both oogenesis and vitellogenesis, suggesting that these two endocrine signals might be cooperative to produce fully grown oocytes. The hormonal mixture effect of JH and insulin on ovarian development was analyzed (Fig. 7). JH II was highly effective in inducing ovarian development. However, ovarian development induced by JH II was not as high as, but its mediation was not enough compared that in control females (Fig. 7a). Addition of insulin significantly enhanced the effect of JH on ovarian development. Decapitated females could develop fully grown oocytes with co-injection of JH II and insulin, exhibiting similar ovarian development like control females (Fig. 7b).

## Discussion

This study investigated the influence of endocrine signals on *M. vitrata* egg development. Insect female reproduction is controlled by JH and ecdysteroids along with nutritional signal [9]. The nutritional signal is mediated by ILPs in egg development of *Drosophila* [26]. According to this general physiological pattern, egg development of *M. vitrata* would also exhibit high dependency on endocrine factors.

There are two ovaries in *M. vitrata*, with each ovary containing four ovarioles. The number of ovarioles per ovary is commonly species-specific. It has great variations across insects, ranging from less than five per ovary in some flies to hundreds per ovary in some grasshoppers [3]. Microscopic analysis of *M. vitrata* oocyte development using fluorescence dyes indicated that its ovariole could be divided into germarium and vitellarium, in which germarium was characterized by previtellogenic oocytes while vitellarium was filled with growing matured oocytes. Like other holometamorphic insects, the ovariole of *M. vitrata* is polytrophic because each oocyte is linked with nurse cells and surrounded by follicular epithelium. In *Drosophila*, oocyte development occurs in germarium from germline stem cells by four cycles of asymmetric cell divisions, in which 15 cells become nurse cells while the remaining cells become oocytes [23]. In the germarium of *M. vitrata* ovariole, cell divisions were detected by BrdU staining and dividing nuclei were observed from DAPI staining, indicating its oocyte development. At the terminal germarium, the oocyte was distinct from nurse cells and surrounded by follicular epithelium. After that, oocytes grew in size probably by accumulating nutrients including Vg from hemolymph. Finally, fully grown oocytes at the proximal ovariole were coated with chorion to be ovulated into the oviduct before oviposition. This is the first detailed analysis of egg development of *M. vitrata* by examining oocyte development and subsequent developmental stages.

*Vg* expression of *M. vitrata* was dependent on larval nutrients, JH, and IIS. Vg protein was specifically detected in female hemolymph of *M. vitrata*. LC-MS/MS analysis of Vg band showed that it was highly matched with other lepidopteran Vg proteins. Its apparent size (approximately 200 kDa) on protein gel was similar to the predicted molecular size (202 kDa) based on Vg gene. This study also identified a VgR of *M. vitrata*. VgR is a member of the low density lipoprotein receptor family that can transport vitellogenin into ovaries to promote ovarian growth and embryonic development [7]. In insects, the only widely accepted ligand of VgR is Vg [35]. During vitellogenesis, Vg is synthesized in the fat body, released into hemolymph [16], and uptaken through VgR of growing oocytes to serve as a nutrient reserve for developing embryo [7]. *Vg* and *VgR* gene expression levels were altered by larval nutrition quality in *M. vitrata*. The effect of larval diet on adult reproduction in *M. vitrata* has been reported in a previous study [36], where different larval diets have resulted in different adult fecundity (109.2 vs. 174.2 eggs laid by each female). In another lepidopteran insect (*Spodoptera exigua*), *Vg* and *VgR* expression levels are also markedly modulated by host nutrients [37]. This can be interpreted by mediation of IIS under nutrient storage in the fat body. In *Drosophila*, fat body can sense amino acids and send a nutritional signal called fat body-derived signal [38]. In response to the fat body-derived signal, insulin-producing cells (IPCs) in the brain produce ILPs to directly or indirectly activate Vg production [39]. JH has been regarded as a main gonadotropin along with 20E and neuropeptides [40, 41]. However, different lepidopteran species vary in JH and 20E dependency according to different reproductive characteristics in terms of the onset of Vg synthesis [40, 42]. In type I insects (*Bombyx mori* [43], *Antheraea yamamai* [44], and *Lymantria dispar* [45–47]), Vg synthesis is mediated by 20E at the last larval or early pupal stage. In type II as seen in *Plodia interpunctella* [48], Vg synthesis is triggered by low 20E titers at pupal stage. In type III, Vg synthesis is independent to 20E as seen in *M. sexta* [15]. In type IV insects including *Heliothis virescens* [8, 49], *Helicoverpa zea* [50], *Pieris brassicae* [51], *Nymphalis antiopa* [52], *Danaus plexippus* [53], *Vanessa cardui* [54], *D. chrysippus* [55], *Pseudaletia unipuncta* [56], and *Spodoptera frugiperda* [57], Vg synthesis is mediated by JH at early adult stage. Thus, *M. vitrata* could be included in the last lepidopteran group because its Vg expression was dependent on JH, but not on 20E. JH II was most effective in inducing *Vg* expression in *M. vitrata*. Most lepidopteran species in general use JH I and JH II [58]. Similar result for *S. exigua* has been obtained in our previous report showing that both JH I and JH II can inhibit metamorphosis of pupae when they are applied to young pupae whereas JH III cannot [59]. In comparison, the hemolymph of *S. litura*, a close taxonomical species to *S. exigua*, has only JH II [60]. These results suggest that endogenous JH of *M. vitrata* is JH II which is a main gonadotropin.

JH stimulated vitellogenesis, but not oogenesis, in *M. vitrata*. Oogenesis was markedly influenced by IIS under diet signal. In *Drosophila*, IIS regulates germline stem cell proliferation [61, 62] and triggers vitellogenesis from the fat body in response to nutritional signal [24, 25]. Thus, ovarian growth is arrested at the previtellogenic stage in *Drosophila* with mutant IIS components [63]. *Chico* (*Drosophila gene* corresponding to insulin receptor substrate) mutant flies display reduced proliferation of follicular stem cells. Their follicles fail to progress to the vitellogenic stage, even in the presence of abundant nutrients [61, 64]. In *M. vitrata*, all four RNAi treatments against IIS components prevented oocyte development, including oocyte differentiation and vitellogenesis. IIS role in stimulating oocyte development was further supported by the observation that addition of a porcine insulin to decapitated females significantly reversed the reduced development of oocytes in *M. vitrata*. Tu et al. [65] have shown that ILP indirectly influences JH biosynthesis through control of JH regulatory neuropeptides. Thus, ILP can have dual positive effect on egg development of *M. vitrata* by activating germline stem cell proliferation and indirectly activating JH synthesis. The dual effect of ILP on previtellogenic and vitellogenic developments suggests a cooperative effect of ILP on JH II because both oogenesis and vitellogenesis could be stimulated by these hormone treatments. Our current study showed that porcine insulin significantly enhanced the effect of JH II on oocyte development. In mosquitoes using 20E as a gonadotropin, ILPs also regulate *Vg* expression indirectly through the regulation of ecdysone synthesis after blood meal [66]. The functional relationship between JH/20E and ILP is well explained using model insects, in which JH/20E via IIS stimulates Vg expression through derepression of FOXO by phosphorylation [67].

## Conclusions

This study determined two endocrine signals of ILP and JH as gonadotropins of *M. vitrata*. These two endocrine signals cooperatively promoted egg development, in which ILP stimulated previtellogenic development by proliferation of germline stem cell in response to fat body-derived signal while JH mediated vitellogenesis by activating *Vg* expression.

## Methods

### Insect rearing

Rearing of *M. vitrata* followed the method described by Jung et al. [68].

### Chemicals

For hormonal assays, JH I (C_18_H_30_O_3_) and JH II (C_17_H_28_O_3_) were purchased from Scitech (Praha, Czech). JH III (C_16_H_26_O_3_), porcine insulin (C_254_H_377_N_65_O_75_S_6_), farnesoic acid (FA: C_15_H_24_O_2_), and 20-hydroxyecdysone (20E: C_27_H_44_O_7_) were purchased from Sigma-Aldrich Korea (Seoul, Korea). Acetone was purchased from Duksan Chemicals (Ansan, Korea).

For immunocytochemistry assays, bromodeoxyribose uridine (BrdU) and fluorescein isothiocyanate (FITC)-tagged phalloidin were obtained from Sigma-Aldrich Korea. 4′,6-diamidino-2-phenylindole (DAPI) was purchased from Thermo Fisher Scientific (Rockford, IL, USA).

### Analysis of ovarian development

Virgin females (1–8 days old) were used. Ovary was dissected in 100 mM phosphate-buffered saline (PBS, pH 7.4) under a stereomicroscope (Stemi SV11, Zeiss, Germany). Ovarioles were separated from the female body and transferred onto slide glass to make them straight. Previtellogenic oocytes were located at the distal region. They had no apparent size increase with well differentiation of nurse cells. Vitellogenic oocytes exhibited apparent size increase in oocytes presumably by accumulation of vitellogenin (Vg). Chorionic oocytes were characterized by chorion formation at the proximal region of ovarioles. Each treatment was replicated with three different females. Total oocyte number was calculated by multiplying the number of oocytes in each ovariole by eight due to the presence of eight ovarioles in a pair of ovaries.

### Analysis of larval nutrient on ovarian development in adults

Based on a standard artificial diet (‘AD’), six other diets were prepared by adding different amounts of main legume components (Additional file 4: Table S1). The resulting seven different diets were fed to L1 for the entire larval feeding period. Each treatment used 30 larvae. Newly molted pupae (< 12 h) were weighed and virgin females at 5 days after emergence were dissected to assess ovarian development by counting oocytes. Randomly chosen 10 females were assessed in each diet treatment.

### Decapitation and hormonal treatment

Newly emerged *M. vitrata* females were decapitated and used for hormonal analysis. For hormonal treatment, decapitated females were injected with 3 μL of hormone or solvent with a microsyringe (Hamilton, Reno, NV, USA). JH I, JH II, JH III, FA, and 20E (in 100% ethanol) were dissolved in acetone (concentration in mg/mL). A porcine insulin powder was dissolved in PBS with 1 M HCl (pH 8.0 adjusted with NaOH). It was then diluted with PBS to obtain desired concentration in mg/mL. All hormonal treatments used a concentration of 1 μg per female.

### RNA extraction, cDNA synthesis, and qPCR

RNA extraction and cDNA preparation followed a method described in Al Baki et al. [27]. Estimation of gene expression levels used qPCR under the guideline of Bustin et al. [69]. PCR conditions were described in Al Baki et al. [27] using forward and reverse primers (Additional file 5: Table S2). Expression of *β-actin* was used as reference because of its relatively stable expression in different tissues of *M. vitrata* [70]. Quantitative analysis was performed using the comparative CT (2^-ΔΔCT^) method [71]. All qPCRs were replicated three times using independent biological samples.

### Fluorescence microscopic analysis

Ovaries from 5 days old virgin females were collected in PBS and separated into ovarioles. Ovarioles were then fixed with 3.7% paraformaldehyde in a wet chamber under darkness at room temperature (RT) for 60 min. After washing three times with PBS, cells in ovarioles were permeabilized with 0.2% Triton X-100 in PBS at RT for 20 min. Cells were then washed three times in PBS and blocked with 5% skim milk (MB cell, Seoul, Korea) in PBS at RT for 60 min. After washing once with PBS, ovarian cells were incubated with FITC-tagged phalloidin in PBS at RT for 1 h. After washing three times with PBS, cells were incubated with DAPI (1 mg/mL) diluted 1000 times in PBS at RT for 2 min for nucleus staining. After washing three times in PBS, ovarian cells were observed under a fluorescence microscope (DM2500, Leica, Wetzlar, Germany) at 200x magnification.

### In vitro organ culture and BrdU incorporation

For in vitro organ culture, ovaries from 5 days old virgin females were collected and cultured in TC-100 insect cell culture medium (Hyclone, Daegu, Korea) containing 10 μM BrdU (Sigma-Aldrich, Seoul, Korea) for 24 h at 25 °C. These ovaries were then fixed, permeabilized, and blocked by the methods described above. After washing ovaries with PBS, cells were incubated with mouse anti-BrdU antibody (BD Bioscience, San Jose, CA, USA) diluted 1:15 in blocking solution for 1 h. After washing three times in PBS, the ovary was then incubated with FITC-conjugated anti-mouse antibody (Sigma-Aldrich, Spruce street, St. Louis, USA) diluted 1:300 in blocking solution at RT for 1 h. After washing three times with PBS, cells were stained with DAPI as described above. These ovarian cells were then observed under the fluorescence microscope.

### SDS-PAGE for vg analysis

Tissues were collected for 10% SDS-PAGE analysis. L5 larval hemolymph was collected and the plasma was separated by centrifugation at 200 x *g* for 3 min. Virgin females and males at 5 days old were selected and used to extract hemolymph and reproductive organs. Hemolymph was collected by PBS injection to adult hemocoel and subsequent suction. These hemolymph samples were then centrifuged at 200 x *g* for 3 min to obtain supernatant plasma. Ovaries and testes were collected by dissection of female and male adults, respectively. Reproductive organs were then ground in PBS and centrifuged at 14,000 x *g* for 3 min to obtain supernatants. All protein samples were quantified by Bradford [72] assay. Each 100 μg protein sample was loaded to 10% SDS-PAGE. After gel running at 125 V constant, separated protein bands were stained with Coomassie brilliant blue and destained with mixture of 50% methanol and 10% acetic acid for 2 h.

### Liquid chromatography-tandem mass (LC-MS/MS) analysis

To confirm Vg from females, its corresponding protein band in molecular size was excised and sent to a proteomic analysis center of Genomine Inc. (Pohang, Korea). After in-gel digestion, the resulting tryptic peptides were analyzed using reversed phase HPLC coupled to an ion trap mass spectrometer (LC-MS/MS) (LCQ Deca XP Plus, Thermo Finnigan, San Jose, CA, USA) using a method of Zuo et al. [73]. Individual spectra from MS/MS were processed using TurboSEQUEST software (Thermo Quest). Generated peak list files were used to query NCBI using MASCOT program (https://pfam.xfam.org). Protein identification used MASCOT probability analysis at scores above 50.

### Statistical analysis

All results were expressed as mean ± standard deviation and plotted using Sigma plot (Systat Software, San Jose, CA, USA). Means were compared by least square difference (LSD) test of one-way analysis of variance (ANOVA) using PROC GLM of SAS program [74] and discriminated at Type I error = 0.05.

## Additional files

Additional file 1: **Figure S1.** Oocyte development of *M. vitrata*. Ovaries from 5 days old virgin female were collected and their ovarioles were separated. Newly dividing cells were specifically recognized by BrdU incorporation (green) while nucleus was stained with DAPI (blue). Cells were observed under a fluorescence microscope. (A) Distal germarial area containing terminal filament and stem cell niche at 200x magnification. (B) Mid-germarial area showing non-oocyte (NOC) and oocyte (OC) at 200x magnification. (C) Proximal germarial area showing previtellogenic oocytes surrounded by follicular epithelium (FE) at 400x magnification. Nurse cells (NC) are neighboring to oocytes (asterisk), indicating polytrophic ovarioles of *M. vitrata. (TIF 5952 kb)*

Additional file 2: **Figure S2.** Domain (A) and phylogenetic (B) analyses of vitellogenin (Vg) of *M. vitrata*. Domains were predicted by Pfam (https://pfam.xfam.org), including Vg amino terminal (Vg-N), domain of unknown function (DUF 1943), and von willebrand factor type D (VWD). Amino acid sequences of Vg were retrieved from GenBank with the following accession numbers: XP_013168895.1 for *Papilio xuthus,* XP_021195456.1 for *Helicoverpa armigera,* NP_001037309.1 for *Bombyx mori,* XP_022836548.1 for *Spodoptera litura,* AOH73254.1 for *Spodoptera exigua,* XP_011555415.1 for *Plutella xylostella,* XP_971398.1 for *Tribolium castaneum,* XP_006616039.1 for *Apis dorsata,* XP_003492277.1 for *Bombus impatiens,* and MG799570 for *Maruca vitrata.* Amino acids were aligned with ClustalW. Phylogenetic analysis was performed using MEGA6. Bootstrapping values were obtained with 1000 repetitions to support branch and clustering. (DOCX 340 kb)

Additional file 3: **Figure S3.** Domain (A) and phylogenetic (B) analyses of vitellogenin receptor (VgR) of *M. vitrata*. Domains were predicted by Pfam (https://www.pfam.xfam.org/), including LDL receptor (LR) and calcium binding EGF (EGF). Amino acid sequences of vitellogenin receptor (VgR) were retrieved from GenBank with the following accession numbers: XP_013181939.1 for *Papilio xuthus,* AGF33811.2 for *Helicoverpa armigera,* XP_022818502.1 for *Spodoptera litura,* AOX13593.1 for *Spodoptera exigua,* NP_001184180.1 for *Bombyx mori,* XP_022125502.1 for *Pieris rapae*, XP_011564499.1 for *Plutella xylostella,* AAC28497.1 for *Aedes aegypti,* XP_016767970.1 for *Apis mellifera,* XP_019847160.1 for *Bactrocera dorsalis*, and MG799569 for *M. vitrata*. Amino acids were aligned with ClustalW and phylogenetic analysis was performed using MEGA6. Bootstrapping values were obtained with 1000 repetitions to support branch and clustering. (DOCX 383 kb)

Additional file 4: **Table S1.** Diet treatment and nutrient composition. (DOCX 15 kb)

Additional file 5: **Table S2.** Primers used in this study for RT-qPCR. (DOCX 16 kb)

## Data Availability

Sequence data supporting the conclusions of this article are included within the article and its additional files. Four dsRNAs specific to IIS component genes are available from YK upon request.

## Abbreviations

20E: 20-hydroxyecdysone
Akt: Serine-threonine protein kinase
CH: Chorionated
FA: Farnesoic acid
FOXO: Forkhead Box O
IIS: Insulin/insulin-like growth factor signal
ILP: Insulin-like peptide
InR: Insulin receptor
IPC: Insulin-producing cell
JH: Juvenile hormone
PV: Previtellogenic
TOR: Target of rapamycin
Vg: Vitellogenin
VgR: Vitellogenin receptor
VT: Vitellogenic
