# Supplementary figures and images for: Insulin signaling mediates previtellogenic development and enhances juvenile hormone-mediated vitellogenesis in a lepidopteran insect, Maruca vitrata
Source: BMC Dev Biol. 2019 Jul 5;19:14. doi: 10.1186/s12861-019-0194-8 (PMC6610926; doi:10.1186/s12861-019-0194-8)

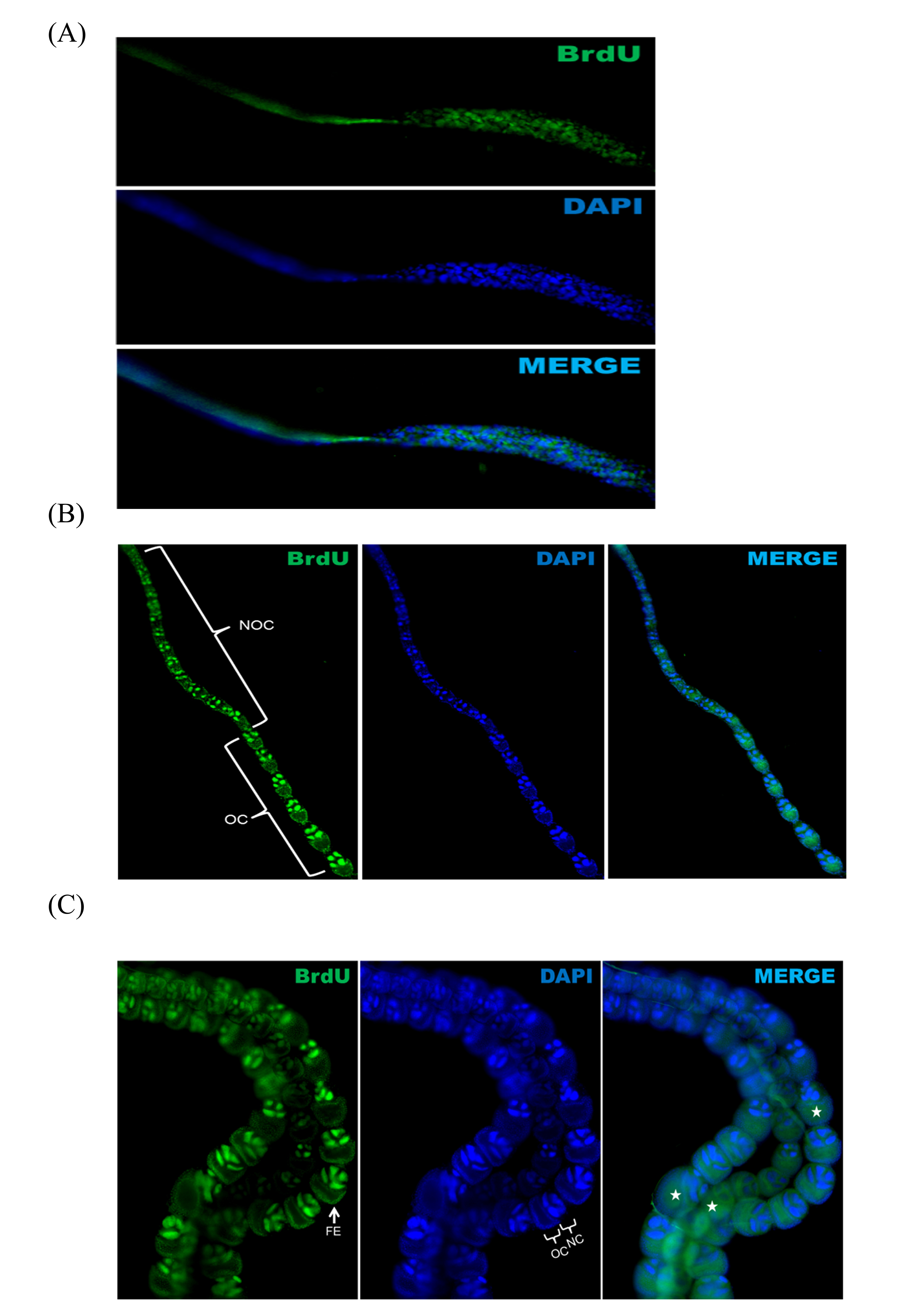

Supplement: Supplementary file 1 — Figure S1. Oocyte development of M. vitrata. Ovaries from 5 days old virgin female were collected and their ovarioles were separated. Newly dividing cells were specifically recognized by BrdU incorporation (green) while nucleus was stained with DAPI (blue). Cells were observed under a fluorescence microscope. (A) Distal germarial area containing terminal filament and stem cell niche at 200x magnification. (B) Mid-germarial area showing non-oocyte (NOC) and oocyte (OC) at 200x magnification. (C) Proximal germarial area showing previtellogenic oocytes surrounded by follicular epithelium (FE) at 400x magnification. Nurse cells (NC) are neighboring to oocytes (asterisk), indicating polytrophic ovarioles of M. vitrata. (TIF 5952 kb) [file 12861_2019_194_MOESM1_ESM.tif]

**A**

**
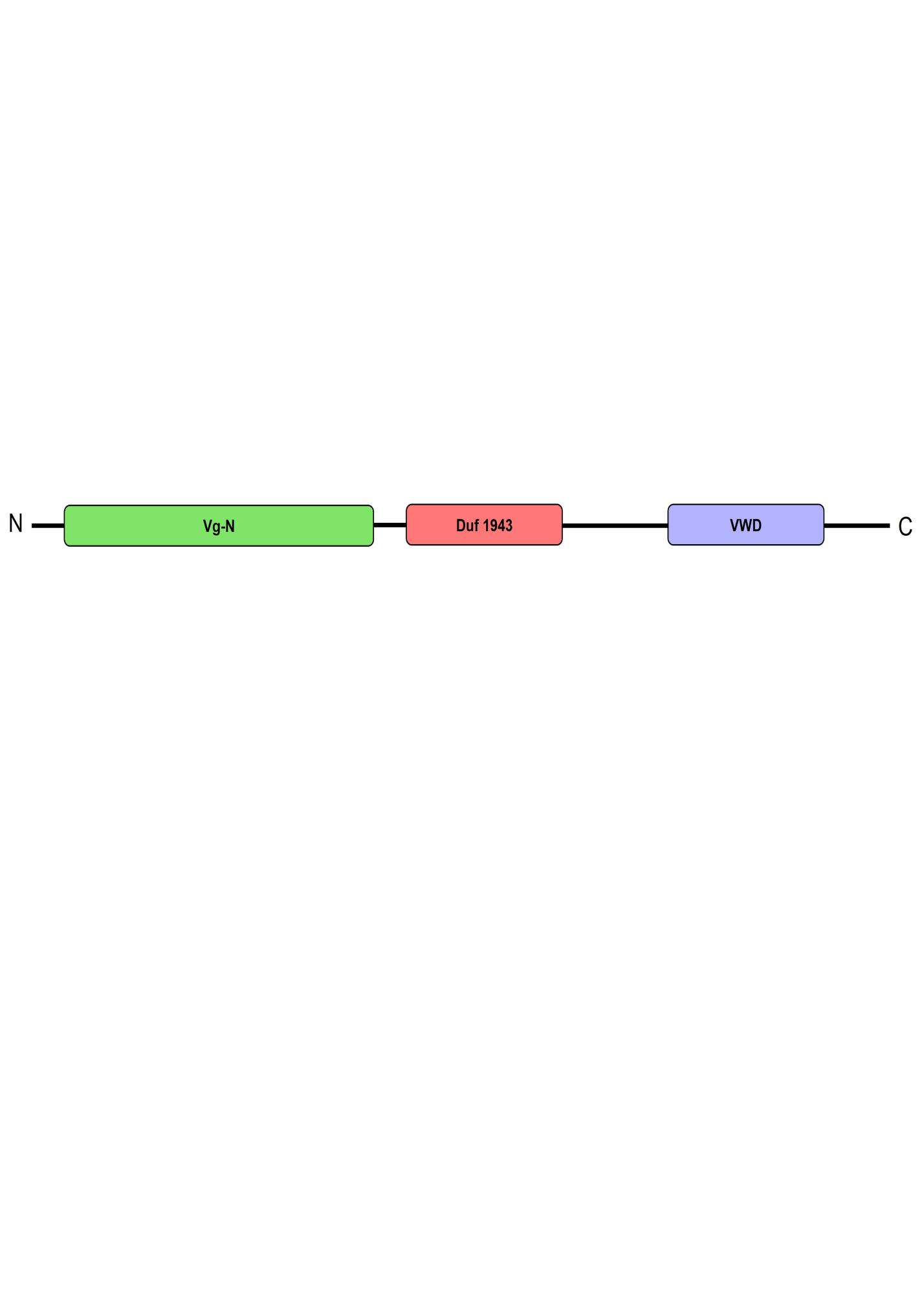
**

**B**


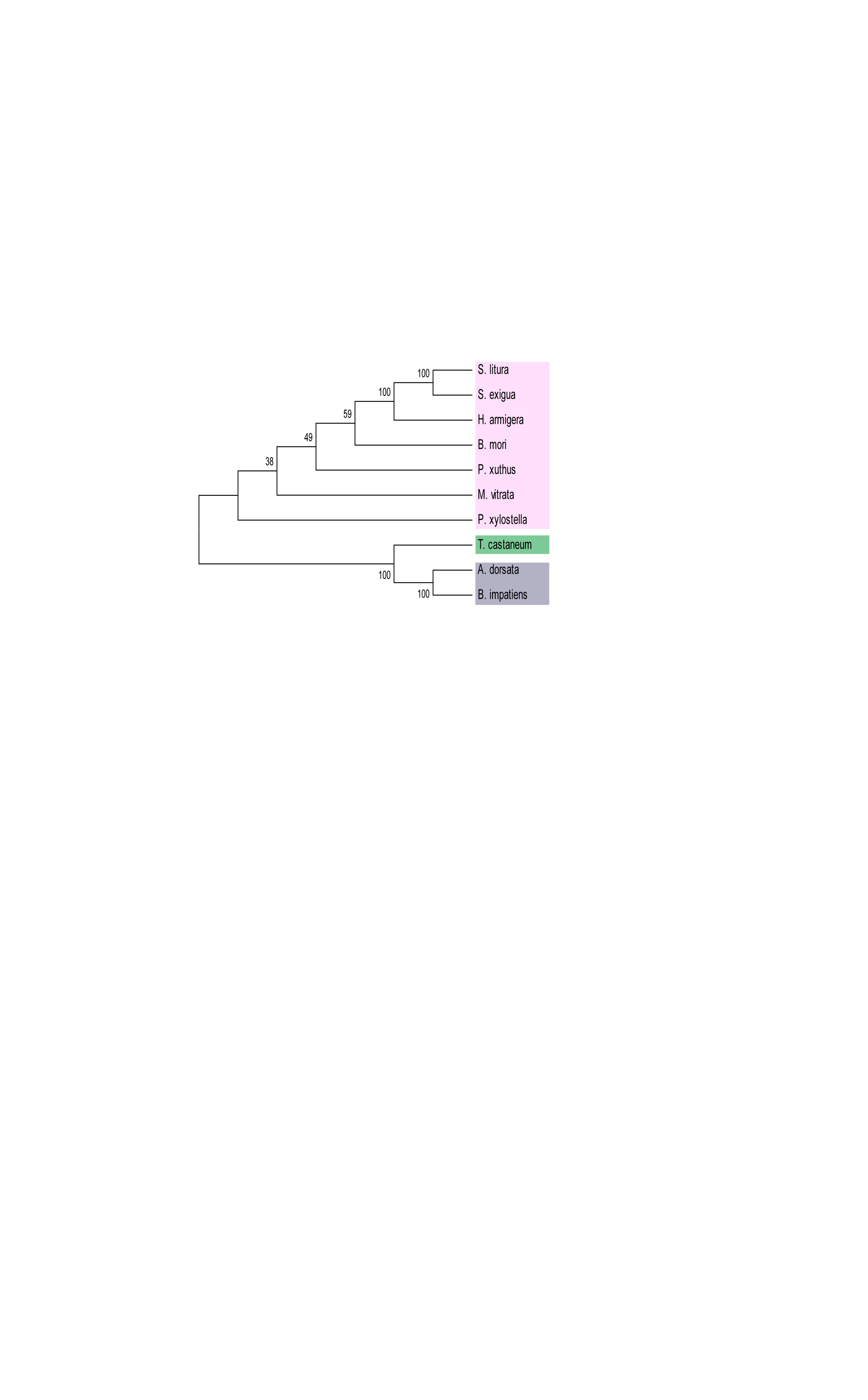


**Fig. S2**

Supplement: Supplementary file 2 — Figure S2. Domain (A) and phylogenetic (B) analyses of vitellogenin (Vg) of M. vitrata. Domains were predicted by Pfam (https://pfam.xfam.org), including Vg amino terminal (Vg-N), domain of unknown function (DUF 1943), and von willebrand factor type D (VWD). Amino acid sequences of Vg were retrieved from GenBank with the following accession numbers: XP_013168895.1 for Papilio xuthus, XP_021195456.1 for Helicoverpa armigera, NP_001037309.1 for Bombyx mori, XP_022836548.1 for Spodoptera litura, AOH73254.1 for Spodoptera exigua, XP_011555415.1 for Plutella xylostella, XP_971398.1 for Tribolium castaneum, XP_006616039.1 for Apis dorsata, XP_003492277.1 for Bombus impatiens, and MG799570 for Maruca vitrata. Amino acids were aligned with ClustalW. Phylogenetic analysis was performed using MEGA6. Bootstrapping values were obtained with 1000 repetitions to support branch and clustering. (DOCX 340 kb) [file 12861_2019_194_MOESM2_ESM.docx]

**A**


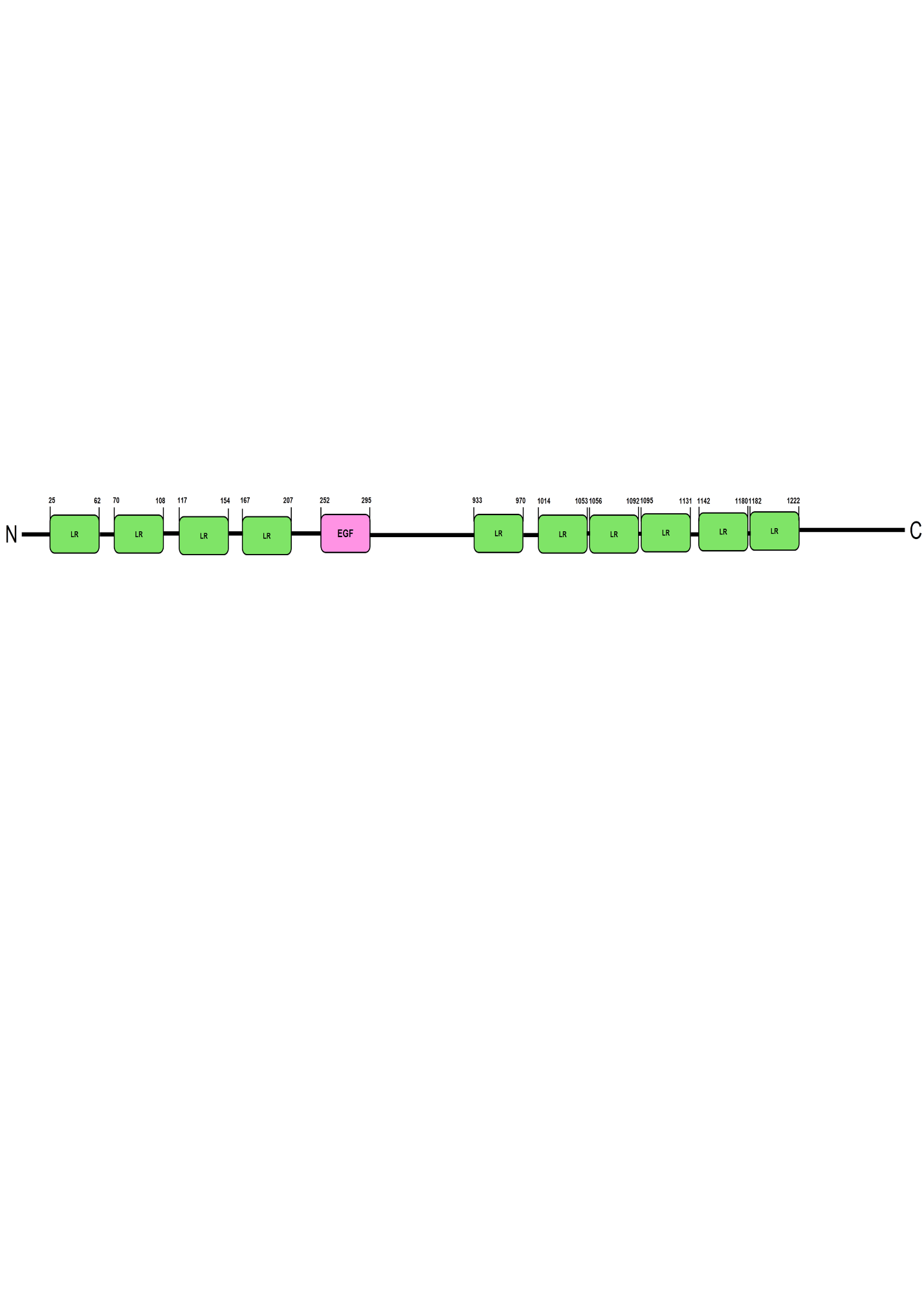


**B**


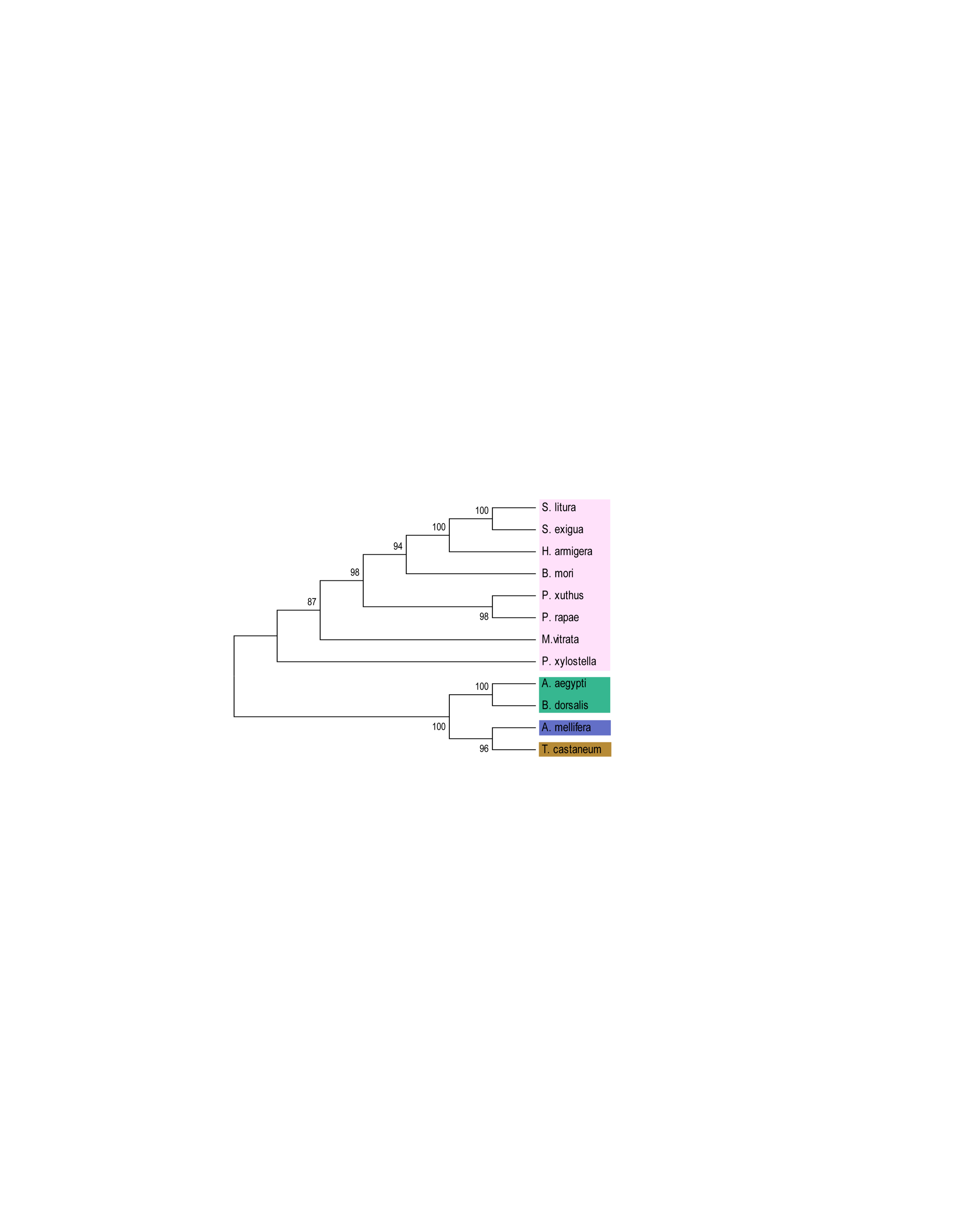


**Fig. S3**

Supplement: Supplementary file 3 — Figure S3. Domain (A) and phylogenetic (B) analyses of vitellogenin receptor (VgR) of M. vitrata. Domains were predicted by Pfam (https://www.pfam.xfam.org/), including LDL receptor (LR) and calcium binding EGF (EGF). Amino acid sequences of vitellogenin receptor (VgR) were retrieved from GenBank with the following accession numbers: XP_013181939.1 for Papilio xuthus, AGF33811.2 for Helicoverpa armigera, XP_022818502.1 for Spodoptera litura, AOX13593.1 for Spodoptera exigua, NP_001184180.1 for Bombyx mori, XP_022125502.1 for Pieris rapae, XP_011564499.1 for Plutella xylostella, AAC28497.1 for Aedes aegypti, XP_016767970.1 for Apis mellifera, XP_019847160.1 for Bactrocera dorsalis, and MG799569 for M. vitrata. Amino acids were aligned with ClustalW and phylogenetic analysis was performed using MEGA6. Bootstrapping values were obtained with 1000 repetitions to support branch and clustering. (DOCX 383 kb) [file 12861_2019_194_MOESM3_ESM.docx]
