# Supplementary material for: Insulin signaling mediates previtellogenic development and enhances juvenile hormone-mediated vitellogenesis in a lepidopteran insect, Maruca vitrata
Source: BMC Dev Biol. 2019 Jul 5;19:14. doi: 10.1186/s12861-019-0194-8 (PMC6610926; doi:10.1186/s12861-019-0194-8)
Supplement: Supplementary file 4 — Table S1. Diet treatment and nutrient composition. (DOCX 15 kb) [file 12861_2019_194_MOESM4_ESM.docx]

**Table S1**. Diet treatment and nutrient composition

| Components | Standard  diet (g) | Adzuki bean diets (g) | | | Cowpea diets (g) | | |
| --- | --- | --- | --- | --- | --- | --- | --- |
|  |  | T1 | T2 | T3 | T1 | T2 | T3 |
| Agar | 13.0 | 13.0 | 13.0 | 13.0 | 13.0 | 13.0 | 13.0 |
| Glucose | 10.0 | 10.0 | 10.0 | 10.0 | 10.0 | 10.0 | 10.0 |
| Vitamin | 5.0 | 5.0 | 5.0 | 5.0 | 5.0 | 5.0 | 5.0 |
| Ascorbic acid | 4.0 | 4.0 | 4.0 | 4.0 | 4.0 | 4.0 | 4.0 |
| Sorbic acid | 1.0 | 1.0 | 1.0 | 1.0 | 1.0 | 1.0 | 1.0 |
| Cellulose | 10.0 | 10.0 | 10.0 | 10.0 | 10.0 | 10.0 | 10.0 |
| Wheat germ | 10.0 | - | - | - | - | - | - |
| Adzuki bean | 20.0 | 105.0 | 50.0 | 20.0 | - | - | - |
| Soybean | 75.0 | - | - | - | - | - | - |
| Cowpea | - | - | - | - | 105.0 | 50.0 | 20.0 |
| Cholesterol | 3.0 | 3.0 | 3.0 | 3.0 | 3.0 | 3.0 | 3.0 |
| β-Sitosterol | 1.0 | 1.0 | 1.0 | 1.0 | 1.0 | 1.0 | 1.0 |
| Methyl-*p*-hydroxybenzoate | 1.5 | 1.5 | 1.5 | 1.5 | 1.5 | 1.5 | 1.5 |
| Aureomycin | 0.5 | 0.5 | 0.5 | 0.5 | 0.5 | 0.5 | 0.5 |
| Fumidil B | 0.4 | 0.4 | 0.4 | 0.4 | 0.4 | 0.4 | 0.4 |
| Water | 800.0 | 800.0 | 800.0 | 800.0 | 800.0 | 800.0 | 800.0 |
