# Supplementary material for: Insulin signaling mediates previtellogenic development and enhances juvenile hormone-mediated vitellogenesis in a lepidopteran insect, Maruca vitrata
Source: BMC Dev Biol. 2019 Jul 5;19:14. doi: 10.1186/s12861-019-0194-8 (PMC6610926; doi:10.1186/s12861-019-0194-8)
Supplement: Supplementary file 5 — Table S2. Primers used in this study for RT-qPCR. (DOCX 16 kb) [file 12861_2019_194_MOESM5_ESM.docx]

| Gene | Primer sequence (5' - 3') | Annealing temperature (°C) | Purposes |
| --- | --- | --- | --- |
| Insulin receptor (InR) | F: GTCGCAACAAGTACGACAGC | 58 | RT-PCR  RT-qPCR |
|  | R: CAGGTAGGTCTTCAGGTCGC |  |  |
|  | F: TAATACGACTCACTATAGGGAGA GTCGCAACAAGTACGACAGC | 58 | RNAi |
|  | R: TAATACGACTCACTATAGGGAGA CAGGTAGGTCTTCAGGTCGC |  |  |
| Protein kinase B (Akt) | F: GAGATACTAACCGCAGCCTTTC | 55 | RT-PCR  RT-qPCR |
|  | R: CACTTACGACACGTTCTCACTATC |  |  |
|  | F: TAATACGACTCACTATAGGGAGA GAGATACTAACCGCAGCCTTTC | 55 | RNAi |
|  | R: TAATACGACTCACTATAGGGAGA CACTTACGACACGTTCTCACTATC |  |  |
| Forkhead box protein O (FOXO) | F: AATCGTTCAGGTAGGCAATCC | 52 | RT-PCR  RT-qPCR |
|  | R: CGCGCCCGAATCCTATAAAT |  |  |
|  | F: TAATACGACTCACTATAGGGAGA AATCGTTCAGGTAGGCAATCC | 52 | RNAi |
|  | R: TAATACGACTCACTATAGGGAGA CGCGCCCGAATCCTATAAAT |  |  |
| Target of Rapamycin (TOR) | F: GCTTTCTTTGGTCTTGAGGAAC | 55 | RT-PCR  RT-qPCR |
|  | R: TCAGCACAACGTCGAACA |  |  |
|  | F: TAATACGACTCACTATAGGGAGA GCTTTCTTTGGTCTTGAGGAAC | 55 | RNAi |
|  | R: TAATACGACTCACTATAGGGAGA TCAGCACAACGTCGAACA |  |  |
| Vitellogenin (Vg) | F: TACTTCGTAGACAGCACACC | 57 | RT-PCR  RT-qPCR |
|  | R: TAAGAAGCGGCGTAGTGTTC |  |  |
| Vitellogenin receptor (VgR) | F: TACTTCGTAGACAGCACACC | 56 | RT-PCR  RT-qPCR |
|  | R: ATCCTCGCGGAGAATGTATC |  |  |
| β-Actin | F: CATCACCATCGGAAACGAAAGG | 52 | RT-PCR  RT-qPCR |
|  | R: ATACTGTGTTGGCGTACAGGTC |  |  |

**Table S2.** Primers used in this study for RT-qPCR
